# The Effectiveness of Tranexamic Acid in Patients With Proximal Femoral Tumor Resection Prosthesis

**DOI:** 10.7759/cureus.10105

**Published:** 2020-08-29

**Authors:** İsmail Burak Atalay, Aliekber Yapar, Coskun Ulucakoy, Emek Mert Duman, Güray Toğral, Recep Ozturk, Bedii Şafak Güngör

**Affiliations:** 1 Orthopedics and Traumatology, Dr. Abdurrahman Yurtaslan Ankara Oncology Training and Research Hospital, Ankara, TUR; 2 Orthopedics, Dr. Abdurrahman Yurtaslan Ankara Oncology Training and Research Hospital, Ankara, TUR

**Keywords:** tranexamic acid, malignant bone tumor, blood reduce, blood transfusion

## Abstract

Aim: The aim of this study is to evaluate the risk of thromboembolic events and amount of postoperative blood loss and transfusion in patients who received preoperative tranexamic acid (TXA) administration in proximal femoral resection and endoprosthesis of proximal femur malignant lesion.

Methods: In this study, the data of 46 patients who underwent extensive resection and proximal femoral tumor prosthesis for proximal femoral bone malignancies were retrospectively reviewed. Patients were divided into two groups according to preoperative 15 mg/kg bolus intravenous administration of TXA. These patients were compared in terms of postoperative blood loss, postoperative bleeding, and transfusion requirements.

Results: There were 46 patients (18 female, 28 male) with a mean age of 60.7±14.7 (19-89) years. Fifteen patients (32.6%) were treated with iv TXA. In the TXA group (46.7%), there was a statistically significant decrease in the need for transfusion compared to the patient group (93.5%) without TXA (p=0.001). Postoperative 24th hour, 48th hour,and total drainage blood loss values were found to be significantly lower in the TXA group (p=0.047, p=0.015, and p=0.019, respectively). There was no thromboembolic event observed.

Conclusion: Because of proximal femoral malignancy, extensive tumor resection and preoperative bolus 15 mg/kg TXA administration in proximal femoral prosthesis surgery significantly decreased the amount of postoperative bleeding and transfusion requirement without increasing the risk of thromboembolic event.

Level of Evidence: Level III - retrospective comparative study.

## Introduction

Resection or amputation are traditional methods of treatment of bone tumors, and wide radical excision forms the basis of surgery for malignant bone tumors. In patients who underwent extremity-sparing surgery, autogenous grafts, vascularized fibula grafts, allografts, endoprosthetic replacement, or alloprosthetic composite can be used for reconstruction. In studies that did not use antifibrinolytic after major surgeries, significant perioperative blood loss ranging from 500 ml to 2000 ml was observed [1-3]. Increased transfusion requirements associated with increased blood loss bring about transfusion-related complications. There is an increasing number of studies that develop methods to reduce perioperative blood loss associated with orthopedic tumor resection. Lysine analogue drugs inhibit the conversion of plasminogen that breaks down fibrin clots to plasmid, thereby reducing the breakdown of clots and creating an antifibrinolytic effect. Tranexamic acid (TXA) is a commonly used lysine analogue drug. As it has an antifibrinolytic effect, it is increasingly used in practice to reduce blood loss. Studies in Europe and the USA have reported that intravenous administration of TXA significantly reduces blood loss and transfusion requirements in the postoperative period [2,3,4]. In the literature, there are many studies reporting the decrease in postoperative blood loss in patients receiving TXA. In addition, it has been reported in the literature that TXA is used in malignancies [5-8]. In a study performed in patients with malignant bleeding, there was no evidence that the use of TXA increased the risk of thrombosis [8].

TXA is routinely administered to patients with malignancy in our clinic by intravenous injection. The aim of this study is to evaluate perioperative blood loss and transfusion needs of patients who underwent tumor resection prosthesis due to malignancy with proximal femur involvement in our clinic compared to the application of TXA. The main hypothesis of this study is that there is a difference between postoperative bleeding amounts between patients who receive TXA and those who do not.

## Materials and methods

This research is an observational descriptive study. The data of patients who met the inclusion criteria for bone malignancies with proximal femoral involvement between January 2018 and June 2019 were reviewed retrospectively in the orthopedics clinic of our hospital, a tertiary education and research hospital specializing in cancer. The patients were divided into two groups as preoperative 15 mg/kg intravenous bolus TXA-administered patients and those who were not administered. When similar studies in the literature were examined, it was observed that the amount of bleeding drained after TXA use in major orthopedic surgeries decreased significantly compared to patients who did not take TXA. In a study by Shen et al. examining the effect of iv TXA use on total postoperative bleeding reduction after total knee arthroplasty, it was found that there was 210 ml less blood loss in the TXA-using group compared to the non-using group [9]. 

According to the sample calculation made using these sample literature data, the ratio of the TXA-using and non-using group in the 80% power to 95% confidence interval was assumed to be 1:2, and the number of samples calculated for TXA was 15 for the using group, 30 for the non-using group and a total of at least 45 patients were planned to be included in the study. The study was completed with a total of 46 patients, 15 of whom were treated with TXA and 31 without treatment.

Ethics committee approval was obtained before the study. Age, gender, body mass index (BMI) values, preoperative hemogram results, blood pressures, perioperative hemodynamic characteristics, treatments, transfusion requirements, postoperative hemogram, and blood loss were obtained from the patients' records. Accordingly, the amount of bleeding drained in the postoperative period of the patients with and without TXA was compared.

The inclusion criteria were patients with wide tumor resection and proximal femoral endoprosthesis between 18 and 80 years of age with proximal femoral malignancies involvement. The control group of the study consisted of patients with the following diseases for which we could not use TXA; inflammatory or autoimmune diseases, thromboembolic disease history, severe anemia, peripheral neuropathy; TXA or contraindication of low-molecular weighted heparin; preoperative anticoagulant drug use to be.

The exclusion criteria were patients with a lack of medical records (six patients) and who have not had regular bleeding follow-up (three patients).

A total of 46 tumor resection prosthesis patients (18 females, 28 males) with a mean age of 60.7±14.7 (19-89) years were included in this study. Seventeen patients had primary bone tumors and 29 had bone metastases. Fifteen patients (32.6%) received intravenous TXA (Table 1).

**Table 1:** Baseline demographics

Parameters (n=46)	
Age, years Mean ± SD Median(min-max)	60.7 ± 14.7 59 (19-89)
Sex, n(%) Male Female	18 (39.1) 28 (60.9)
Operative Side, n(%) Left Right	22 (47.8) 24 (52.2)
Diagnosis, n(%) Primary bone tumor Metastasis	17 (37.0) 29 (63.0)
Use of tranexamic acid, n(%) Yes No	15 (32.6) 31 (67.4)

In both groups, intraoperative blood loss was evaluated by blood volume and whole blood and sponge count to evaluate TXA efficacy. These two groups were then evaluated by calculating post-operative 24th hour, 48th hour, and total drain blood loss values. Intraoperative transfusion, postoperative transfusion, and total transfusion were evaluated from the medical records of the patients.

The postoperative regimen included a compression bandage used until discharge, weight-bearing of extremity 24 hours after surgery, and drains were removed 48 hours after surgery. All patients received the same postoperative anticoagulation regimen (4000 IU low molecular weight heparin once daily by subcutaneous injection).

## Results

There was no statistically significant difference between TXA-using and non-using patients in terms of age, BMI, gender distribution, operation side, diagnosis, preoperative hemoglobin value (Hb), preoperative international normalized ratio (INR), type of anesthesia, and staying time in the hospital (p>0.05) (Table 2). In addition, early postoperative exitus rates were similar in both groups (p=1.000). No postoperative deep venous thrombosis (DVT) and pulmonary thromboembolism (PTE) were observed.

**Table 2:** Baseline demographics and the laboratory parameters in the two groups. INR: international normalized ratio

N=46	Use of tranexamic acid			
	Yes (n=15)	No (n=31)		P
Age, years Mean ± SD Median(min-max)	54.5±17.2 55 (19-77)		63.7± 12.5 63 (38-89)	0.4031
Sex, n(%) Male Female	7(46.7) 8(53.3)		11 (35.5) 20 (64.5)	0.6852
Operative Side, n(%) Left Right	6(40) 9(60)		16 (51.6) 15 (48.4)	0.6712
Diagnosis, n(%) Primary bone tumor Metastasis	6(40) 9(60)		11 (35.5) 20 (64.5)	1.0002
BMI, kg/m^2^ Mean ± SD Median(min-max)	26.8±5.7 25.7(19.1-40.4)		26.2±4.4 25.9 (14.0-34.3)	0.7343
Pre-operative Haemoglobin, g/dl Mean ± SD Median(min-max)	11.8±1.4 11.9(9.9-15.3)		11.7± 1.9 11.8 (8.3-17.6)	0.8723
Pre-operative PT(INR) Mean ± SD Median (min-max)	1.03±0.13 1.03(0.8-1.3)		1.04± 0.12 1.0 (0.8-1.4)	0.9763
Type of anesthesia, n(%) General Spinal	11(73.3) 4(26.7)		18 (58.1) 13 (41.9)	0.4972
Postoperative exitus, n(%) Yes No	2(13.3) 13(86.7)		5 (16.1) 26 (83.9)	1.0002
Hospitalization time, days Mean ± SD Median(min-max)	6.3±2.5 6 (2-12)		7 ± 2.8 8 (2-14)	0.1781
^1^Mann-Whitney U test ^2^Chi-Square Test ^3^Independent Sample T-Test				

In Table 3, perioperative transfusion and blood loss values ​​of TXA-using and non-using groups were compared. While there was no significant difference between the distribution of transfusion patients and the amount of transfusion in the intraoperative period, the need for transfusion was significantly lower in the TXA-using group (46.7%) than in non-using group in the postoperative period (93.5%) (p=0.001). There was no statistically significant difference between the TXA-using group and the non-using group in terms of postoperative transfusion amount (p=0.477). Intraoperative blood loss values ​​were similar in both groups (p=0.108). Postoperative 24th hour, 48th hour, and total drainage blood loss values ​​were found to be significantly lower in the TXA-using group respectively than non-using group (p=0.047, p=0.015, and p=0.019, respectively). It was found that total blood loss was statistically significantly different in TXA-using and non-using groups and it was lower in the TXA-using group than the non-using group (p=0.017).

**Table 3:** Transfusion and postoperative blood loss in the two groups.

N=46	Use of tranexamic acid		
	Yes(n=15)	No(n=31)	P
Total Transfusion (Number of patients), n(%) Yes No	12 (80) 3 (20)	30 (96.8) 1 (3.2)	0.0951
Total Transfusion (n=12/30), ml Mean ± SD Median(min-max)	1387.5± 929.4 1125(450-3600)	1500 ± 671.9 1800(450-3150)	0.4332
Intra-operative Transfusion (Number of patients), n(%) Yes No	10 (66.7) 5 (33.3)	27 (87.1) 4 (12.9)	0.1271
Intra-operative Transfusion (n=10/27), ml Mean ± SD Median(min-max)	1080± 677.5 900(450-2250)	900 ± 353 900(450-1800)	0.7032
Postoperative Transfusion (Number of patients), n(%) Yes No	7 (46.7) 8 (53.3)	29 (93.5) 2 (6.5)	0.0011
Postoperative Transfusion (n=7/29), ml Mean ± SD Median (min-max)	771.4± 219.6 900(450-900)	713.8± 307 450(450-1350)	0.4772
Intraoperative blood loss,ml Mean ± SD Median (min-max)	1026.7± 727.9 950(200-2600)	1290.3± 490.1 1100(750-2400)	0.1082
Postoperativedrainageat24h,ml Mean ± SD Median (min-max)	433.3± 327.1 400(100-1200)	517.7± 201.1 500(250-1120)	0.0472
Postoperative drainage at 48h,ml Mean ± SD Median (min-max)	286.7± 311.9 200(50-1300)	362.9± 168.8 300(100-800)	0.0152
Postoperative total amount of drainage, ml Mean ± SD Median (min-max)	720±600 500(200-2500)	880.7± 348.0 800(400-2000)	0.0192
Amount of total blood loss,ml Mean ± SD Median(min-max)	1746.7± 1233.2 1500(500-5000)	2170.9± 742.2 2100(1150-4400)	0.0172
Chi-SquareTest ^2^Mann-Whitney U test			

## Discussion

In recent years, the importance of TXA use in reducing the amount of bleeding in specific operations of orthopedics such as hip and knee arthroplasties has been proven in many studies [1-4]. Patients with malignant tumors located at the bone are at higher risk of bleeding and thromboembolic events than primary arthroplasties. The aim of this study was to investigate the effect of preoperative intravenous administration of TXA in patients with proximal femur primary tumor or metastasis who underwent proximal femur resection and proximal femur resection prosthesis in terms of intraoperative and post-operative bleeding and perioperative thromboembolic events. It was observed that this application did not cause a decrease in the amount of intraoperative bleeding but caused a significant decrease in post-operative bleeding amount and did not increase thromboembolic events.

There are few studies in the literature about the use of TXA to reduce bleeding in malignant tumor surgical procedures [5-8]. Montroy et al. published a meta-analysis in which lysine analogues examined the efficacy and safety of cancer patients. According to this analysis, the administration of a lysine analogue reduces both blood loss and need for transfusion, and also lysine analogues do not increase the risk of thromboembolic events in cancer patients [8].

Although TXA is effective in many different types of surgical procedures, differences in fibrinolytic response by surgical type have shown that the optimal regimen for TXA administration is probably procedure-specific [10]. This suggests that TXA should be investigated in different types of surgeries. In the literature review, there was no study investigating the effect of TXA administration on bleeding amounts, transfusion requirement, and the risk of thromboembolic events in the surgery of malignant tumors located on the limb bones.

When some other cancer-related studies were examined, in a review investigating the effect of TXA administered during radical cystectomy for bladder cancer, TXA did not provide significant benefit compared to placebo [6]. In another study, it was reported that the use of TXA in head and neck cancers was not beneficial in reducing bleeding rates [7]. In another study examining the effect of TXA use in vertebral metastasis surgery, no significant benefit was reported in the amount of bleeding. In another study, it was reported that TXA reduced bleeding rates in Complex Skull Base Neurosurgical Procedures including malignant tumor surgeries. When all these studies were evaluated, it was stated that TXA did not increase thromboembolic events [6,7,11,12].

The proximal femur is a common location for the localization of primary and metastatic malignant tumors. Resection of a malignant tumor located in the proximal femur and salvage of the limb by subsequent reconstruction is the basis of treatment for proximal femoral malignancies. This reconstruction can be a proximal femoral endoprosthesis (tumor prosthesis) or an allograft-prosthesis combination. Reasons such as early loading on extremity, cost-effectiveness, and easy availability have made arthroplasty the primary option in limb salvage surgery [13-15].

It is known that TXA should be applied preoperatively in order to obtain maximum effect on hip arthroplasties. In periodical studies or analyses, there was no difference in blood loss between patients who received a single dose TXA bolus preoperatively and patients receiving additional TXA doses [10]. In this study, we investigated preoperative single-dose administration for our hip localized operations. 

There are several opinions in the literature regarding the dose of TXA intravenous administration. In their prospective cohort study in 2019, Mannová et al. showed that doses of 10 mg/kg and 15 mg/kg decreased bleeding in total hip and knee arthroplasty [16]. Similarly, Watts et al. proved the effectiveness of 15 mg/kg systemic TXA in hemi- and total hip arthroplasty after a femoral neck fracture [17]. In our study, we applied 15 mg/kg systemic TXA.

There were some limitations of this study. First of all, this was a retrospective analysis and the number of patients was relatively low. In addition, although it was an advantage that the same one specialist performed all the surgeries, the present study included the findings of a single center Another limitation is that the type and size of the tumor, which can affect the amount of bleeding, was not specified. Prospective larger studies with higher patient numbers are needed in the future.

## Conclusions

As a result, preoperative 15 mg/kg TXA administration as bolus significantly reduces postoperative bleeding amount and transfusion requirements in wide tumor resection and proximal femoral tumor prosthesis surgery due to malignant tumor located in the proximal femur. This procedure did not increase the risk of thromboembolic events in patients with bone cancer, compared to the group of patients without TXA administration.
